# Gynaecologists’ views on the management of Vaginal Vault Prolapse: A qualitative study

**DOI:** 10.12669/pjms.38.3.5215

**Published:** 2022

**Authors:** Omaema Al-Baghdadi, Christian Barnick, Garima Srivastava, Hassan M Elbiss

**Affiliations:** 1 Omaema Al-Baghdadi, Tawam Hospital, Al Ain, UAE. Surrey University, Guildford, United Kingdom; 2 Christian Barnick, Homerton University Hospital, Homerton Row, London, United Kingdom; 3 Garima Srivastava, Homerton University Hospital, Homerton Row, London, United Kingdom; 4 Hassan M Elbiss, Departments of Obstetrics and Gynaecology, College of Medicine and Health Sciences, UAE University, Al Ain, UAE

**Keywords:** Vault prolapse, Qualitative research, Sacrocolpopexy, Laparoscopy

## Abstract

**Objective:**

This study examined gynaecologists’ experience and views on the management of vaginal vault prolapse (VVP) using laproscopic sarcocolpopexy (LSCP) versus open sarcocolpopexy (OSCP).

**Methods:**

In a qualitative study conducted at the University of Surrey and Homerton University Hospital, UK, from 2016 to 2017, semi-structured interviews were conducted with 15 consultants experienced in minimal access surgery or urogynecology. Interviews were recorded and transcripts were analyzed using the qualitative description (QD) approach.

**Results:**

Eight broad themes emerged: VVP management, LSCP for management of VVP, OSCP and vaginal surgery with or without mesh use in VVP management, laparoscopic training and support as well as surgeons’ attitude towards LSCP. All participants acknowledged the importance of LSCP in the management of post-hysterectomy VVP as benefits outweighed risks in their view. OSCP was considered suitable in very specific circumstances. Vaginal surgery could be an excellent alternative to OSCP bearing in mind long-term efficacy and sexual activity in young women. Most participants agreed with national recommendations to avoid use of mesh in vaginal surgery for VVP and expressed the view that it should be done in specialised centres by trained surgeons who do such operations.

**Conclusions:**

This study showed that the acceptability of LSCP was dependent on participants’ experience and consideration of the balance between patient’s goals and potential risks. It provides useful guidance for future large-scale projects.

## INTRODUCTION

Genital prolapse, a common condition among women over 50 years of age, severely affects their life quality leading to withdrawal from social activity due to urinary, bowel and sexual dysfunction.^1^ Vaginal vault prolapse (VVP) is the main long-term problem following total-hysterectomy.^2^ The chance of undergoing surgery for prolapse is expected to increase with the continuing ageing of the population.^3^

The management of VVP involves pelvic floor physiotherapy, vaginal pessary and vaginal surgery including abdominal, vaginal and laparoscopic procedures to reinstate the normal anatomy including operations such as sacrospinous fixation, colpocliesis and sacrocolpopexy.^4^ The optimal surgical approach remains a considerable challenge and a controversy. Sacrocolpopexy, an abdominal surgical procedure for VVP that involves attaching a mesh between the vaginal vault and sacrum was first described as an open laparotomy.^5^ Open abdominal sacrocolpopexy (OSCP) has been viewed as the gold-standard procedure,^6^ being found to be superior to vaginal sacrospinous fixation.^7^ OSCP takes longer to perform, has a longer recovery time and is more expensive.^7^ Laparoscopic sacrocolpopexy (LSCP), first described in 1992,^8^ has evolved enormously with decreased morbidity, faster recovery and comparable outcomes.^6^,^7^

LSCP is gaining acceptance as the primary treatment for VVP. However, as longer time is required to train surgeons to be skilled in advanced-laparoscopy, gynaecologists are still cautious in using LSCP as an alternative to the traditional approach. Although surgical preferences and techniques for sacrocolpopexy worldwide have been reported to be similar.^9^ there are some geographical variations in LSCP and OSCP.^6^,^7^ It is surprising that none of the studies available have looked at the surgeons’ values, opinions and clinical experience. Finding out from surgeons themselves how acceptable they find LSCP could help identify areas that could explain the lack of consensus about the preferred management of VVP. Difference in views and attitudes is best explored by qualitative research.

We investigated the reasons why gynaecologists are reluctant to adopt LSCP as the standard management of VVP despite the advancement of minimal-invasive approaches in gynaecology.

## METHODS

After obtaining ethical and R&D (FTFHMS-217-15, R&D No 1653, 17 June 2016) approvals from the University of Surrey and Homerton University Hospital, UK, this qualitative study^10^,^11^ conducted in 2016-2017 used semi-structured interviews of participants experienced in post-hysterectomy VVP management in the UK. Informed consent was obtained from all participants. The interviews were conducted either face-to-face or via telephone. They were audio recorded and transcribed. Qualitative description (QD) method was used to gain an honest knowledge of professionals’ experiences for tailoring clinical interventions via analysis of the interview transcripts to examine gynaecologists’ acceptability of LSCP, their views about other surgical approaches (OSCP and vaginal surgery with or without mesh use) and the potential impact on their patients.

Participants were identified from regional and national professional society conferences. Overall, 18 consultant gynaecologists experienced in minimal-invasive procedures or uro-gynaecology were approached with the participant information sheet for making a voluntary contribution. All responded to the invitation to take part in the study (100%). Consultant-level participants skilled in the management of VVP following total hysterectomy were included because it requires experience to advice women with VVP. Those with less than two-year experience at consultant level or with no experience in VVP management or minimal access surgery were excluded. Due to participant’s availability and time constraints, 15 participants who were able to complete the interview were recruited into the study (83.3%). All participants gave informed consent.

Sample size was according to the National Centre for Research Methods, which states: “a small number of cases or persons is extremely valuable and represent adequate numbers for a research project. This is accurate for studying hard to access people. Therefore, a relatively small number between six and twelve may be enough to give us insight”.^16^ Interviews were started and continued until data became repetitive and the analysis reached saturation. Interviews were scheduled to coincide with the participant availability and permitted time. Wherever possible, interviews were conducted, face-to-face, in a private room; participants were given the option of a telephone interview if this was more suitable. This flexibility was considered necessary for avoiding bias. The confidentiality of participants in the study was preserved by keeping personal details and contact information separate from interview data in accordance with the UK Data Protection Act. Data were collected through semi-structured interviews to ensure high quality and genuine respondent data, and to minimize bias. Interviews were digitally recorded (Olympus VN-732PC) for transcription, ranging 15-54 min in duration.

An interview topic guide was designed based on published literature and expert opinion. It comprised of open-ended questions within a semi-structured format to explore gynaecologists’ general experience and personal views of LSCP and their standard management of post-hysterectomy VVP. This allowed the investigator to suggest the main topics for discussion and participants to offer additional comments. Then the interview progressed to ask specifically about the obstacles and expectations of LSCP as the standard management of post-hysterectomy VVP. Open-ended interview techniques were used to encourage comprehensive and diverse responses without the researcher’s guidance. The interview agenda initially consisted of the following board areas: general information, experience of LSCP, OSCP, vaginal surgery with or without mesh in VVP, views towards training in laparoscopy and LSCP, acceptability of LSCP from their own perspective; cost, unit and work environment, and attitudes towards LSCP.

The recorded and transcribed data were interpreted by two researchers (a consultant gynaecologist and research-design expert). The QD approach explored gynaecologists’ experience and acceptability of LSCP for VVP management with respect to “why”, “how” and “what” questions about views and worries. Therefore, with its mainly inductive approach qualitative analysis was used for problem identification, theory formation and concept development. Commencing after the first five interviews, the interview data were organized and coded into themes manually, which involved identifying and highlighting emergent themes that could be considered particularly important to the interviewee. This process allowed the development of a working analytical framework where thematic codes were grouped together into categories for use in subsequent transcripts. This required judgment in retaining the original meanings and ‘feel’ of the interviewees’ words. For internal reliability a third researcher independently sampled and reassessed the QD analyses carried out.

## RESULTS

Of the total of 15 interviews conducted, nine were face-to-face and six were via telephone. There were eleven males (73.3%). The mean age was 55.86 years (range 39-65). Three were retired consultants. The duration of experience was on average 14.4 years (range 2-25). Five of the interviewed consultants were laparoscopic surgeons, five were uro-gynaecologists and five both. All surgeons were trained in the UK and 12 (80%) had attended short term courses in Europe, Asia and USA. All participants had laparoscopic experience and their practice of minimal access surgery (MAS) was on average 64.3% of their surgical work (range 20-90%). Regarding LSCP, three consultants didn’t do the procedure (two stopped after a period of use), 12 (80%) did it regularly with annual average of ten cases (range 4-40). About OSCP, eleven (73.3%) consultants didn’t do the open surgery; only four (26.6%) did it on a regular basis with an average of seven cases annually (range 4-10). Vaginal surgery was done by all consultants but five didn’t do it for VVP; annual average 47.5 cases of SSF (range 5-150). Mesh use vaginally was only deployed by three (20%) (two used modified tapes and one bio-meshes) in around 5-50 cases per year. Eight board themes and sub-themes that emerged from analysis of all interviews are shown in Table-I.

**Table-I:** Themes and subthemes from interviews conducted in a qualitative study concerning gynaecologists’ views on the management of vaginal vault prolapse.

Main theme	Sub theme
Vulvovaginal prolapse management	Surgeon’s treatment of choice
	Patient selection
	Quality of life
Laproscopic sacrocolpopexy for management of vulvovaginal prolapse	Surgeon’s opinion
	Surgeon’s treatment of choice
	Patient selection surgery
	Benefits of surgery
	Limitations of surgery
	Surgical Training
	Evidence based medicine
Open sacrocolpopexy for management of vulvovaginal prolapse	Surgeon’s opinion
	Patient selection for surgery
	Benefits of surgery
	Limitations of surgery
Vaginal surgery for management of vulvovaginal prolapse	Surgeon’s opinion
	Patient selection for surgery
	Benefits of surgery
	Limitations of surgery
Mesh use in management of vulvovaginal prolapse	Surgeon’s opinion
	Benefits of mesh use
	Limitations of mesh use
	Patient’s judgement
	Patient’s counselling
Training in the United Kingdom	Surgeon’s opinion
	Surgeon’s learning curve
	Situation of training
	Limitations to training
Support to do laproscopic sacrocolpopexy	Work culture
	Cost factor
	Equipment and technology
Surgeons’ attitude towards laproscopic sacrocolpopexy	Surgeon’s background
	Lack of agreement
	Stimulating though

These wide-ranging themes were identified using QD till data saturation. Participants acknowledged the importance of LSCP in the management of post-hysterectomy VVP as benefits outweighed the risks in their view. While LSCP was considered an “excellent approach” and “standard” by twelve surgeons; three participants considered it complex and thought it was not a safe procedure to offer it as first line. All participants considered OSCP to be a highly morbid procedure that was a good option for women with severe symptomatic VVP and recurrent prolapse as well as in situations when surgeons need to convert to laparotomy following failed LSCP. Vaginal approach was considered the “general choice” in VVP management. LSCP as a treatment of VVP was evaluated for benefits and limitations, barriers to success, the future of surgery and the available research. Concerning benefits associated with LSCP, all agreed that it had quick recovery, short hospital state, and value for young fit sexually active women.

Six participants appraised the good visualization, especially in difficult cases with high BMI and intra-abdominal adhesions. Most of the interviewees agreed on the fact that it was “wrong to give ladies serious morbidity for something that is a QoL issue”. They considered women with previous surgeries and potential intra-abdominal adhesions, high BMI, not fit for MAS, anaesthesia risk, and serious medical co-morbidities as high-risk women for an abdominal approach. Other group of surgeons considered high BMI women and abdominal adhesions as better for LSCP. With respect to evidence, participants clarified the lack of comparative studies: “Patients are difficult to match. It’s difficult to get large numbers unless you do multicenter studies. If you do multicenter studies, you are looking at lots of different surgeons. Different surgeons have slightly different techniques and even if you standardize the techniques, they have different levels of ability and different types of procedure, whether it’s laparoscopic or vaginal.”

Advantages and disadvantages of mesh use in the management of VVP abdominally (LSCP/OSCP) or vaginally was considered critical in-patient counselling. Medicolegal issues related to mesh use was conveyed with recommendations to refer patients to tertiary centers. Participants’ vision of training in laparoscopy and LSCP in the UK shed light on the learning process as well as the future of training and the limitations faced. All surgeons believed it to be the main barrier to the evolution of LSCP in the UK. Most agreed training was self-motivated that needed hands-on learning first based in wet-labs and animals, and then finally in humans. LSCP was an advanced procedure that needed advanced skills, which meant that it need time to learn, appropriate theatres and equipment besides good trainer and assistant. Participants also opined critically regarding workplace, support and finance, and their impact on MAS and access to LSCP in the UK.

## DISCUSSION

The demand for treatment of VVP, a long-term problem following total-hysterectomy, has increased in the recent years due to ageing population. It is a well-known condition including in low-middle income countries.^12^-^15^ This research explored experienced gynaecologists’ opinions and acceptability of LSCP as the standard option in the management of post-hysterectomy VVP. Published research and studies in the literature largely focus on outcomes and modalities rather than examining surgeons’ perspectives. To our knowledge there was no qualitative research about the standard approach to manage women with VVP at the time of undertaking the work. This study obtained original data exploring surgeons’ expectations and responses to information on LSCP and other approaches to VVP management. It considered whether LSCP approach influenced VVP management by exploring participants’ views about the laparoscopic approach compared to others approaches, and questioned whether additional areas of targeted training could benefit surgeons to practice such approaches in the future.

Undertaking the QD approach allowed themes to emerge and gave the opportunity to explore concepts, opinions and experiences from collected data. Most of the themes resonated with published quantitative research. Thus, qualitative research enriched the evidence base through the explanation of the professional and personal impact of LSCP approach and other methods. Using semi-structured interviews was an effective means of identifying issues important to surgeons, supported by a sample size adequate for valid qualitative results. Our manuscript complied with a majority of the items of the COREQ reporting checklist.^16^ Being the first qualitative study examining surgeons view about a novel issue, our findings merit consideration.

### Limitations

One limitation was interviewee time constraint. The duration of experience of the participants was variable, a feature that adds to the spectrum of our observations. The consultants were mostly experienced in laparoscopic procedures, so possibly their attitudes were biased in favor of this technique over other approaches. Additionally, as participants were selected from scientific courses, this may impact on generalisability of findings. Another limitation is the disproportionately low female consultant participation (4, 26.6%). A final limitation of the study could be that by taking part in the study the involved surgeons had already expressed their acceptance of such surgery and they wanted to validate their views. Eliciting data through semi-structured interviews relies heavily on the rapport between researcher and participant. The surgeons were identified by the researcher and this combined with the satisfactory sample size and the in-depth analysis produced findings descriptive of a wider sector of gynaecologists.

LSCP’s recent popularity may be related to the decrease in use of mesh vaginally.^17^ There has been a decrease in mesh use vaginally from 25% to 2%.^18^ As a treatment of choice, some consider LSCP to be better than vaginal approach especially in young sexually active women, but this view was not universal in our study. Although LSCP and OSCP have similar outcomes,^19^ more than half the participants considered LSCP as a better option and one rated OSCP higher. The current opposition among some gynaecologists was likely because they were still cautious to use it due to the lack of advanced-laparoscopy skills. One of the published studies reported on the lack of structured curricula; inadequate faculty expertise, and limited time were the most important obstacles.^20^ Evidently, most of the published reports on LSCP were small retrospective and comparative-cohort studies.^21^,^22^ Application and maintenance of an advanced surgical technique is a challenge to practicing consultants and their patients. Future research, considering the importance of patient and public engagement,^23^ would also benefit from evaluating women’s experience of the LSCP approach.

## CONCLUSION

Implementation of a new surgical technique is an ever-present challenge to practicing surgeons and their patients. Laparoscopy is one of these innovative technologies that offers several potential benefits above traditional open or vaginal procedures. This first qualitative study provides new information concerning gynaecologists’ views on the management of VVP and LSCP as the standard approach in this situation. The clinical significance of our findings are that LSCP was recognized as the standard “first line” management of VVP when SCP was indicated. It was viewed as having great value for young sexually active women and as having a high satisfaction rate among patients.

### Authors’ Contribution:

**OAB** and **HME** conceived and designed the study and performed analysis and editing of manuscript and both are responsible and accountable for the accuracy or integrity of the work.

**CB** and **GS** contributed to data collection and manuscript writing, and all authors did review and final approval of the manuscript.
